# Design and Simulation of Air-Breathing Micro Direct Methanol Fuel Cells with Different Anode Flow Fields

**DOI:** 10.3390/mi12030253

**Published:** 2021-03-02

**Authors:** Huichao Deng, Jiaxu Zhou, Yufeng Zhang

**Affiliations:** 1 School of Mechanical Engineering and Automation, Beihang University, Beijing 100191, China; denghuichao@buaa.edu.cn; 2 MEMS Center, Harbin Institute of Technology, Harbin 150001, China; yufeng_zhang@hit.edu.cn

**Keywords:** micro direct methanol fuel cell, simulation, MEMS, serpentine flow field, mass transport

## Abstract

The design of the anode flow field is critical for yielding better performance of micro direct methanol fuel cells (µDMFCs). In this work, the effect of different flow fields on cell performance was investigated by the simulation method. Compared with grid, parallel and double-serpentine flow fields, a single-serpentine flow field can better improve the mass transfer efficiency of methanol and the emission efficiency of the carbon dioxide by-product. The opening ratio and channel length also have important effects on the cell performance. The cells were manufactured using silicon-based micro-electro-mechanical system (MEMS) technologies and tested to verify the simulation results. The experimental results show that the single-serpentine flow field represents a higher peak power density (16.83 mWcm^−2^) than other flow fields. Moreover, the results show that an open ratio of 47.3% and a channel length of 63.5 mm are the optimal parameters for the single-serpentine flow field.

## 1. Introduction

Micro direct methanol fuel cells (µDMFCs) have attracted much attention in portable and mobile electronic products such as notebook computers and mobile phones due to their high efficiency, high energy density, room-temperature operation and simple structure [1,2,3,4,5,6]. The current collector, as a major component of µDMFCs, is responsible for transport of reactants, structural support for membrane electrode assembly (MEA) and current collection. Therefore, the design of an anode flow field on the current collector is a critical factor for increasing µDMFCs performance [7,8].

Many efforts have focused on improving the cell performance by changing the anode flow field design. Flow field parameter optimization and new pattern design were considered to be effective methods to improve cell performance. The open ratios (ratio of perforation area to current collector area) and channel size (channel length, width and depth) have been shown to be the key factors that influence the cell performance. The effects of channel patterns and open ratios (ratio of perforation area to current collector area) on the performance of passive µDMFCs were investigated by experiments and simulations in [9]. Results indicate that the vertical stripe pattern (VSP) is preferred for anodes, and the best performance had an open ratio of 45.6%. The open ratios of the anode and cathode current collectors on the power density of passive µDMFCs were also studied in [10]. The best output (3.14 mWcm^−2^) was scored with a lower open ratio (34%) on both sides with a methanol concentration of 2 M. The behavior of the micro-scale effect in µDMFCs was investigated through both simulations and experiments. The maximum power density (27.1 mWcm^−2^) was scored at the optimal feature size of 0.6 mm and the aspect ratio of 2:1 [11]. In addition, the channel cross-sectional flow field was optimized experimentally. A passive micro direct methanol fuel cell was fabricated with both rectangular and trapezoidal cross-sectional geometry at the anode and cathode using the micro-electro-mechanical system (MEMS) technology fabrication technique. According to results from [12], the power density (6.64 mWcm^−2^) of trapezoidal cross-section channels was nearly two times higher than the power density (3.9 mWcm^−2^) of rectangular cross-section channels at a 7 M methanol concentration. A serpentine channel with a non-uniform cross-section was designed and analyzed in [13]. Numerical results show that non-uniform converging designs enhance the peak power generation performance by as much as 12% at high-current-density operations. In order to improve the performance of µDMFCs, a series of new pattern designs were proposed along with optimization of the flow field parameters [14,15,16,17,18,19].

Parameter optimization and new pattern design anode flow fields have great effects on the performance of µDMFCs. Different flow fields result in different pressure distributions. The pressure difference between adjacent flow channels of the ribs affects the convection and diffusion of methanol in the diffusion layer, changes the distribution of methanol, and affects the performance of µDMFCs. In addition, the pressure difference increases the mass transfer rate of methanol molecules and covers the emission of carbon dioxide. Based on the above considerations, we investigated different anode flow fields of self-breathing µDMFCs. The model with different flow fields was built to analyze the effect of flow field parameters on the performance of µDMFCs. The simulation results show that the single serpentine design gave a better performance and open ratios and channel lengths affect the anode flow field structure. Meanwhile, the silicon-based self-breathing µDMFCs were fabricated to verify the simulation results by MEMS technology. The experimental results agree well with the simulation results.

## 2. Computational Model

### 2.1. Flow Field Design

**Figure 1:**
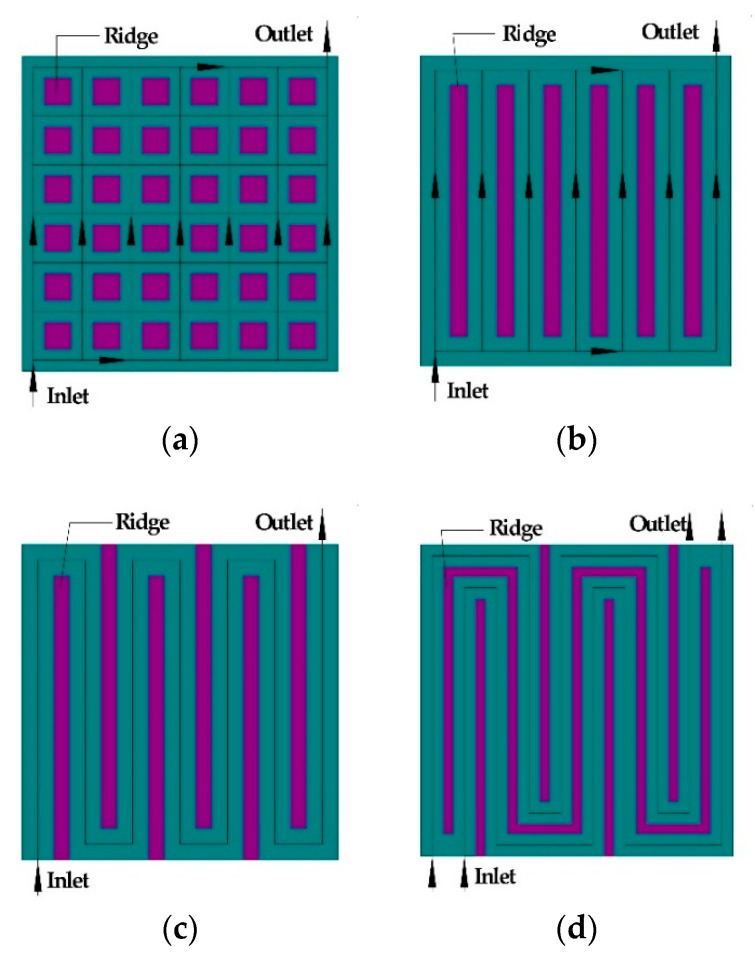
Design of the anode flow fields: (**a**) grid flow fields, (**b**) parallel flow fields, (**c**) single-serpentine flow fields, and (**d**) double-serpentine flow fields.

This paper mainly considers the influence of the anode flow field on cell performance. Therefore, the cathode current collectors are perforated collectors with the same structure. To investigate the effects of anode flow field configurations on the cell performance at the same conditions, four types of flow fields including grid, parallel, single-serpentine and double-serpentine flow field were designed with approximately the same active area and open ratio as shown in Figure 1. The active area and open ratio were set to around 64 mm^2^ and 73.0%, respectively. Other structure parameters are shown in Table 1.

For single-serpentine flow fields, the influence of channel open ratios on cell performance was analyzed by variations of the channel and support ridge widths. The respective open ratios of the four types of single-serpentine flow fields with the same active areas were 29.1%, 47.3%, 60.6% and 73.0%, and other parameters are shown in Table 2.

In addition, four single-serpentine flow fields with different channel lengths and the same open ratios (47.3–47.8%) were designed to study the effects of channel lengths on cell performance, as shown in Table 3. The four channel lengths (47.32, 63.50, 79.60 and 96.03 mm) were realized by modifying the channel widths and the channel numbers (5, 7, 9 and 11). 

### 2.2. Model Description and Assumption

Two factors have significant direct effects on the cell performance for the anode of µDMFCs. Firstly, the speed of methanol mass transport in the diffusion layer impacts the process and efficiency of the electrochemical reaction. Secondly, CO_2_ blocking in the anode negatively influences the methanol mass transport. Therefore, efforts to design the anode flow fields will contribute to improving the cell performance. Thus, we built a three-dimensional steady-state multi-field coupled model for the anode of µDMFCs to investigate the mass transport, including anode flow channels, an anode diffusion layer and an anode catalyst layer. Several assumptions for simplification are introduced in this model:

(1) All processes in the µDMFCs are under steady-state conditions.
(2) The average hole transfer model is applied in the diffusion layer.
(3) The catalyst layer is simplified as a plane without thickness.
(4) The CO_2_ generated at the anode dissolves in water completely, and its influence is ignored.
(5) The methanol density in the anode flow channel is considered as a constant and it belongs to the incompressible flow.
(6) The influence of gravity is ignored.

### 2.3. Governing Equations

(1) Navier–Stokes (N-S) Equation

The momentum transfer of the fluid in the flow channel can be described by the Navier–Stokes (N-S) equation. The influence of CO_2_ can be ignored because the methanol density in the anode flow channel can be considered as a constant. The flow of the methanol in the flow channels is considered to be laminar flow and it is continuous. The momentum of the flow comes from the pressure difference ∇p. The N-S equation can be expressed as follows:

(1) $$\rho \frac{\partial u}{\partial t}+\left(\rho u\cdot \nabla \right)u=-\nabla p+\nabla \cdot \tau +F,$$

(2) ∇·u=0,

(3) $$\tau =\eta \left(\nabla u+{\left(\nabla u\right)}^{T}\right),$$

where ρ is the density of the methanol solution, u represents the flow velocity of the liquid, p is the liquid pressure, τ is the liquid viscid pressure tensor, F represents the external force, and η represents the liquid viscosity coefficient.

(2) Diffusion-Convection Equation

In this model, the influence on fluid that is caused by CO_2_ is neglected, so the mass transfer within the flow channel only occurs between the methanol and water. The diffusion in the flow channel can be described by Fick’s law, and the velocity of the convection can be achieved from the N-S equation. Fick’s law can be expressed as follows:

(4) −D∇c=R,

where D represents the diffusion coefficient of methanol in water, c is the concentration of the methanol, and R represents the reaction rate. The velocity vector in the flow channel is described by the N-S equation and the velocity vector in the diffusion layer is described by Darcy’s law. The mass transfer equation in the flow channel is thus obtained:

(5) ∇·(−D∇c)=R−u∇·c.

The diffusion coefficient of methanol in the diffusion layer is

(6) $$D=D_{eff}\cdot {\left(\varepsilon \right)}^{1.5},$$

(7) $$D_{eff}=D_{ref}\cdot \exp\left(2436\left(\frac{1}{353}-\frac{1}{T}\right)\right),$$

where $D_{ref}$ is the diffusion coefficient of methanol in water at the temperature of 353.15 K, ε represents the absolute permeability of the porous medium, and T is the working temperature of the fuel cell.

(3) Darcy’s Law

In the anode diffusion layer, the momentum of the methanol solution convection comes from the liquid pressure difference and can be described by Darcy’s law:

(8) $$\nabla \cdot \left(\rho \left(-\frac{\kappa }{\eta }\nabla p\right)\right)=Q_{darcy},$$

(9) $$u=-\frac{\kappa }{\eta }\nabla p,$$

where κ represents the absolute permeability of the porous medium, and $Q_{darcy}$ is the source term.

(4) Ohm’s Law

The electron conduction and the distribution of the electron conduction potential in the anode diffusion layer can be described by Ohm’s law:

(10) $$\nabla \left(-\sigma_{l}\nabla \phi_{l}\right)=-S_{a}\cdot i_{a}.$$

The proton conduction and the distribution of the electron conduction potential in the anode diffusion layer can be described as

(11) $$\nabla \left(-\sigma_{i}\nabla \phi_{i}\right)=S_{a}\cdot i_{a},$$

where $\sigma_{i}$ is the effective electronic conductivity, $\varphi_{i}$ is the anode electric potential, $S_{a}$ represents the surface area, and $i_{a}$ represents the anode current density.

(5) Butler–Volmer (B-V) Equation

The anode current density generated by the methanol oxidation in the catalyst layer can be described by the B-V equation:

(12) $$i_{a}=i_{0}\cdot \exp\left(0.5F\frac{eta}{\left(R\cdot T\right)}\right)\cdot \left(\frac{c}{c_{ref}}\right)\cdot \left(c\geq 0\right),$$

where $i_{0}$ represents the exchange current density, F is the Faraday constant, eta represents the overvoltage, and $c_{ref}$ is the reference methanol concentration.

### 2.4. Boundary Conditions

(1) N-S Equation

All the walls in the flow channel are solid except for those of the inlet and outlet, that is to say, the fluid has no movement in the wall. The fluid enters the flow channel at a certain velocity due to the external influence, and the boundary condition for the inlet velocity is

(13) $$n\cdot u=u_{0},$$

where $u_{0}$ represents the liquid feeding rate. As the outlet is exposed to air, so the pressure at the outlet is set as the standard atmosphere:

(14) $$p=p_{0},$$

where $p_{0}$ represents the standard atmosphere.

(2) Diffusion-Convection Equation

The methanol flow into the flow channel through the inlet at the concentration of $c_{0}$, and the boundary condition at the inlet is

(15) $$c=c_{0}.$$

As the concentration of the methanol is uncertain, it is set as the convection flux, which means the diffusion concentration of methanol in the direction of the unit vector is zero. The convection flux is uncertain and its boundary condition is

(16) n·(D∇c)=0.

(3) Darcy’s Law

The walls are all considered insulated and symmetrical. There is a pressure gradient at the boundary face of the interface of the flow channel and the diffusion layer. This pressure gradient can be expressed as below:

(17) $$\frac{\nabla p}{\nabla x}=\frac{\partial p}{\partial n},$$

The velocity is approximately:

(18) $$-n\cdot u=\frac{\left(\frac{\kappa }{\eta }\right)\nabla p}{1\left[mm\right]}.$$

(4) B-V Equation

The mass transfer of the methanol in the catalyst layer is a flux:

(19) $$-n\cdot N=N_{0},$$

(20) N=−D∇c+cu,

(21) $$N_{0}=-\frac{i_{a}}{\left(6\cdot F\right)}.\;$$

(5) Ohm’s Law

The internal inward normal current density $J_{0}$ at the interface of the diffusion layer and the catalyst layer is set as

(22) $$-n\cdot J=J_{0}=-i_{a}.$$

Set the electrical potential energy at the flow channel polar plate as $V_{cell}$:

(23) $$V=V_{cell}.$$

Other surfaces are considered insulated.

### 2.5. Solution Procedure

In the study, the above simulation was implemented in COMSOL Multiphysics^®^ 5.5 (COMSOL, Stockholm, Sweden). COMSOL Multiphysics^®^ 5.5 is a multi-physics simulation software using the finite element method. The governing equations and boundary conditions are implemented using built-in modules (Secondary Current Distribution, Transport of Diluted Species and Darcy’s Law) and custom functions. A structured hexahedral mesh was generated by swept and mapped methods. The given boundary conditions are inlet velocity and pressure. The default steady-state solver was used to solve the model. Other parameters used in the simulation are given in Table 4.

## 3. Simulation Results and Discussion

### 3.1. Analysis of Four Anode Flow Fields

**Figure 2:**
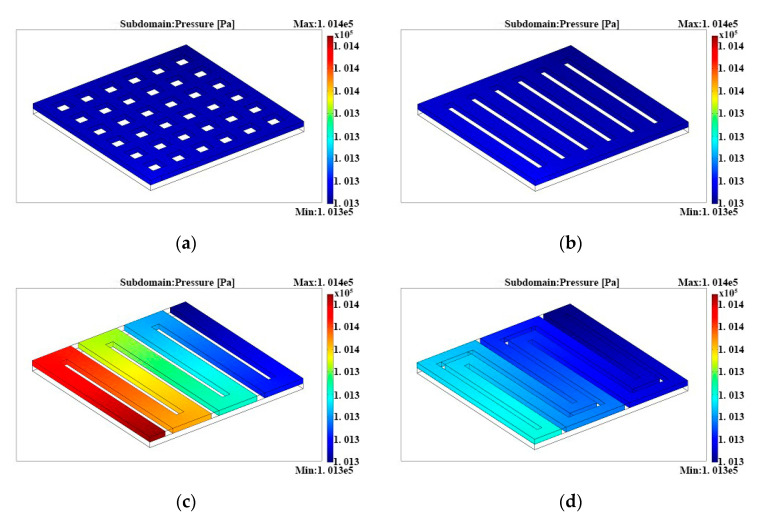
Pressure distributions in (**a**) grid flow fields, (**b**) parallel flow fields, (**c**) single-serpentine flow fields, and (**d**) double-serpentine flow fields.

Figure 2 shows the pressure distributions of four anode flow fields. It was clear that the pressure difference of the inlet and outlet decreased in the following sequence: single-serpentine, double-serpentine, parallel, and grid flow fields, which were 74.39, 30.16, 9.58 and 8.74 Pa, respectively, according to the calculation. It was known that the working precondition of µDMFCs was the methanol flow in anode flow fields. The larger the pressure gradient, the faster and more uniformly the liquid flowed, which was not only propitious to increase the transport efficiency of reactants in the diffusion layer, but also favorable to the exclusion of resultants. In that case, a single-serpentine flow field was the first priority.

**Figure 3:**
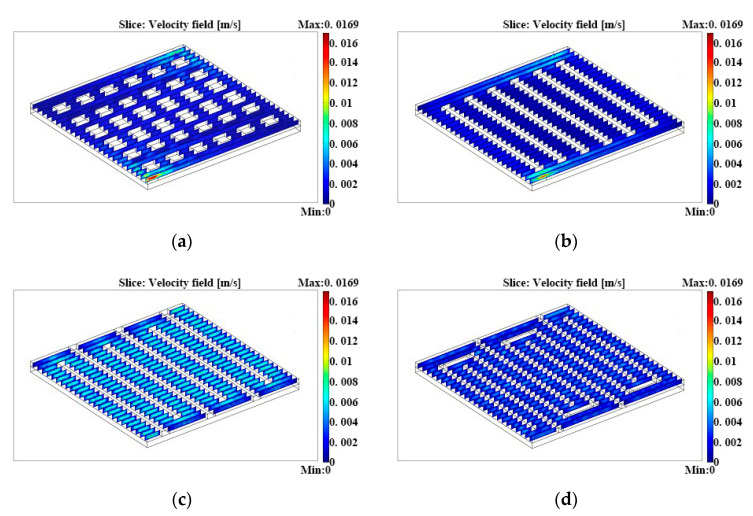
Flow rate distributions in (**a**) grid flow fields, (**b**) parallel flow fields, (**c**) single-serpentine flow fields, and (**d**) double-serpentine flow fields.

Figure 3 illustrates the velocity distributions in four anode flow fields. For the grid flow field and parallel flow field, the flow rate was rather fast at the inlet. However, it had a sharp decrease when the methanol solutions flowed into the channels and stagnant fluid appeared in a large region, which brought an uneven distribution of reactants as well as degradation of flow channel utilization. Furthermore, it had a considerable impact on the exhaust of resultants and heat, which would further cause CO_2_ to block the channel and local over-heating. The above factors could absolutely decrease the lifetime and performance of µDMFCs. However, for single-serpentine and double-serpentine flow fields, the flow rate was even in most of the flow channels, except that there might be a small quantity of stagnancy at the corner of the flow passageway. The respective average flow rates were 0.0094 and 0.0055 m/s, which were more favorable than those of the grid and parallel flow fields.

**Figure 4:**
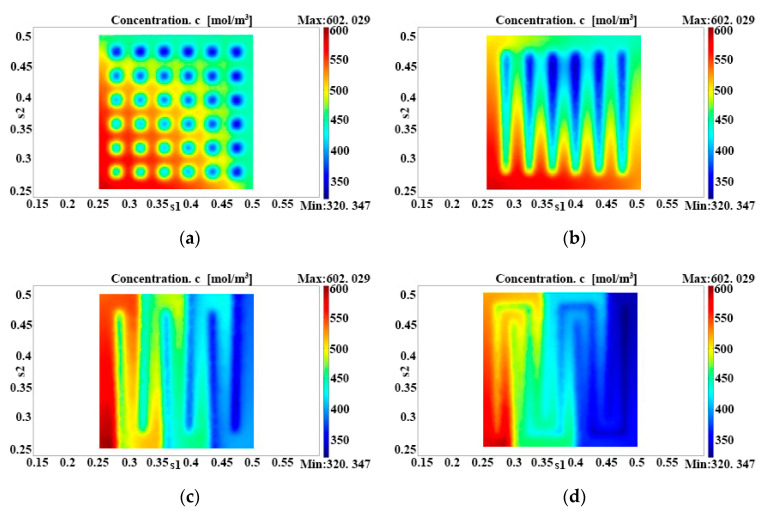
Methanol concentration distributions at the catalyst layers under (**a**) grid flow fields, (**b**) parallel flow fields, (**c**) single-serpentine flow fields, and (**d**) double-serpentine flow fields.

Figure 4 shows the methanol concentration distributions at the catalyst layer for four anode flow fields. Based on the calculation, the respective maximum methanol concentrations were 586.58, 585.29, 602.03 and 590.93 mol/m^3^, and the respective minimum values were 353.27, 336.11, 339.04 and 320.35 mol/m^3^. The single-serpentine flow field could help the catalyst layer obtain the highest methanol concentration that the methanol transport could provide. However, the high pressure of the single-serpentine flow field could also result in the increment of methanol concentration differences. The peak concentrations in the other flow fields were not quite different from each other, and the difference was smallest in the grid flow field. As the diffusion layer directly contacted the support ridge of the flow channels, methanol molecules could enter the bottom of the support ridge by cross motion. Therefore, the methanol concentration in the support ridge, which corresponds to the catalyst layer (the dead mass transport area under the support ridge), was considerably lower than that in the corresponding flow field, which might cause unevenness of concentration distributions. In fact, the methanol molecule transport velocity at the diffusion layer under the support ridge could be enhanced by increasing the pressure difference of neighbor flow channels so that the concentration difference could be reduced.

By solving Equation (12) in the model, we conclude that the highest current densities in the catalyst layer of single-serpentine, double-serpentine, grid and parallel flow fields were 2910.11, 2903.72, 2986.76 and 2931.67 A/m^2^, respectively. Clearly, the single-serpentine flow field was the best in that case. Compared with the other three flow fields, the single-serpentine flow field could guarantee the uniform distributions and high-efficiency mass transport of anode reactants as well as the effective discharge of the products; all of these advantages made the single-serpentine flow field best fit the anode of µDMFCs.

### 3.2. Different Open Ratios of Single-Serpentine Flow Fields

**Figure 5:**
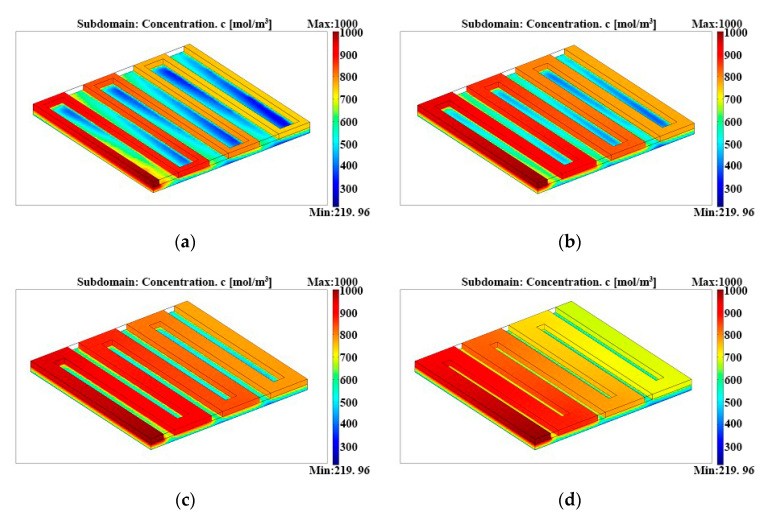
Methanol concentration distributions in the anode single-serpentine flow fields with different open ratios: (**a**) open ratio = 29.1%, (**b**) open ratio = 47.3%, (**c**) open ratio = 60.6%, and (**d**) open ratio = 73.0%.

Figure 5 illustrates the methanol concentration distributions in the single-serpentine anode flow fields with four different open ratios, and the inlet methanol concentrations all set as 1000 mol/m^3^. It is clearly seen that the methanol concentration in the single-serpentine flow field with an open ratio of 73.0% is the largest, with a difference of more than 300 mol/m^3^ between the concentration of the inlet and outlet. However, the dead mass transfer field at the bottom of the support ridge was most distinctive in the anode diffusion layer, with an open ratio of 29.1% under the lowest concentration of 219.96 mol/m^3^. In addition, it was likely to cause the concentration polarization. With the same single-serpentine flow field, we concluded that the wide support ridge could result in the dead mass transport field of reactants, which makes the methanol mass transport hard in the diffusion layer under the ridge. On the other hand, the methanol concentration distributions would be not uniform with a narrow support ridge, and they would also reduce the cell performance. Therefore, we found the best open ratio for anode flow fields to balance the cons and pros. As shown in Figure 5, methanol concentration distributions in anode flow fields were more uniform when the open ratio was 47.3% and 60.6%, and it was in favor of the stability of µDMFCs.

**Figure 6:**
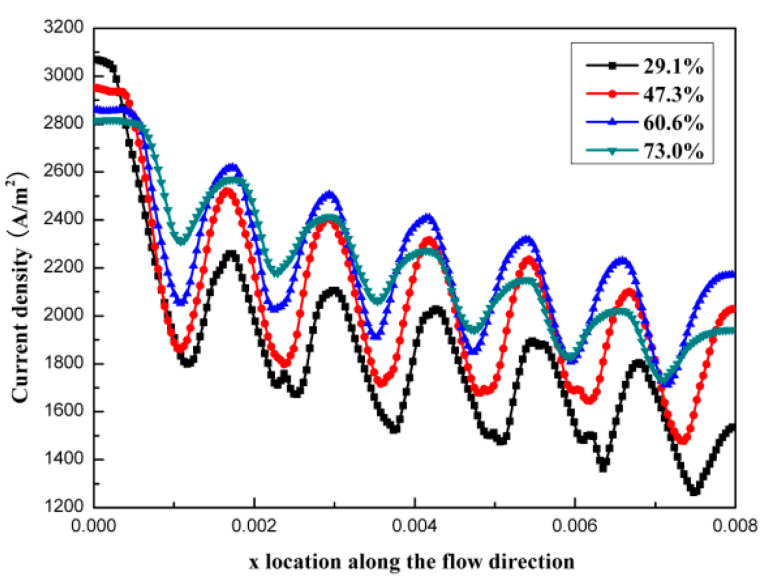
Current density distributions at the catalyst layers along the x direction under the conditions of different open ratios.

Figure 6 shows the current density distributions at the catalyst layers along the x direction under the conditions of different open ratios. According to Figure 6, we could observe that the current density at x = 0 with an open ratio of 29.1% was the highest with a value of 3068.12 A/m^2^. However, the current density at x = 0.008 with the same open ratio was the lowest (only 1263.97 A/m^2^). Comparatively, the current densities were more uniform with open ratios of 60.6% and 73.0%. Therefore, it was favorable to increase the utilizations of reactants and catalysts, while it was not easy to have the concentration polarization.

### 3.3. Different Channel Lengths of Single-Serpentine Flow Fields

**Figure 7:**
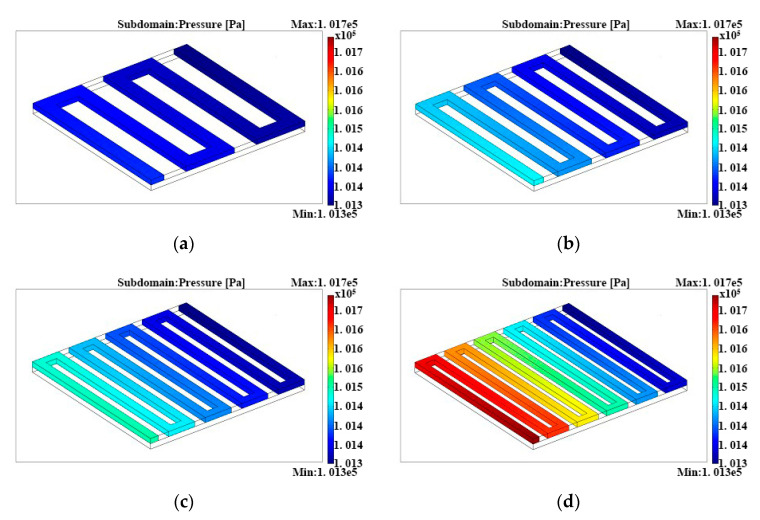
Pressure distributions in the anode serpentine flow fields with different channel lengths: (**a**) channel length = 47.32 mm, (**b**) channel length = 63.50 mm, (**c**) channel length = 79.60 mm, and (**d**) channel length = 96.03 mm.

Figure 7 shows the pressure distributions in the serpentine flow fields with different channel lengths. As shown in Figure 7, the pressure difference of the inlet and outlet increase gradually with the increment of channel lengths. Based on the above discussion, the pressure drop in the flow channels would contribute to the liquid flow, mass transfer of reactants and discharge of products. Therefore, the channel length of 96.03 mm could help the fuel cell achieve the best performance. However, the channel width would be reduced accordingly with an increment of the channel length. For instance, the channel with a length of 96.03 mm could only be 0.33 mm wide. In this way, the anode products CO_2_ blocking the channel will become larger, which not only brings about a decrease of the performance of the fuel cell but also increases the power loss of the external device for liquid feeding.

**Figure 8:**
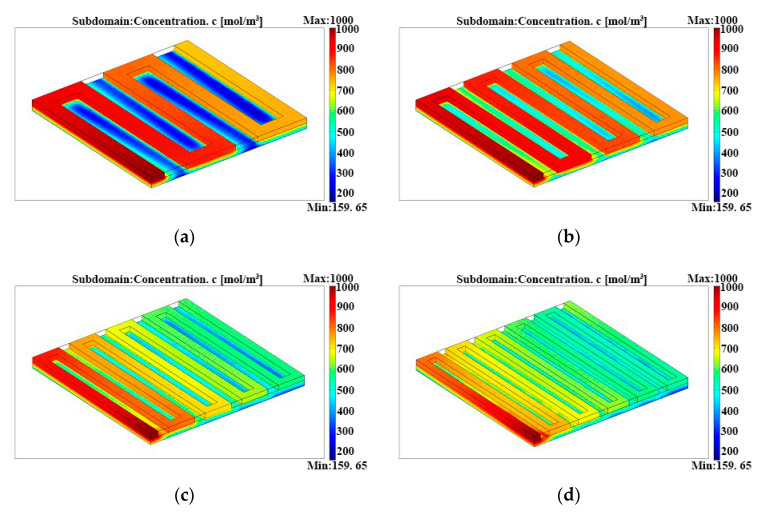
Methanol concentration distributions in the anode serpentine flow fields with different channel lengths: (**a**) channel length = 47.32 mm, (**b**) channel length = 63.50 mm, (**c**) channel length = 79.60 mm, and (**d**) channel length = 96.03 mm.

**Figure 9:**
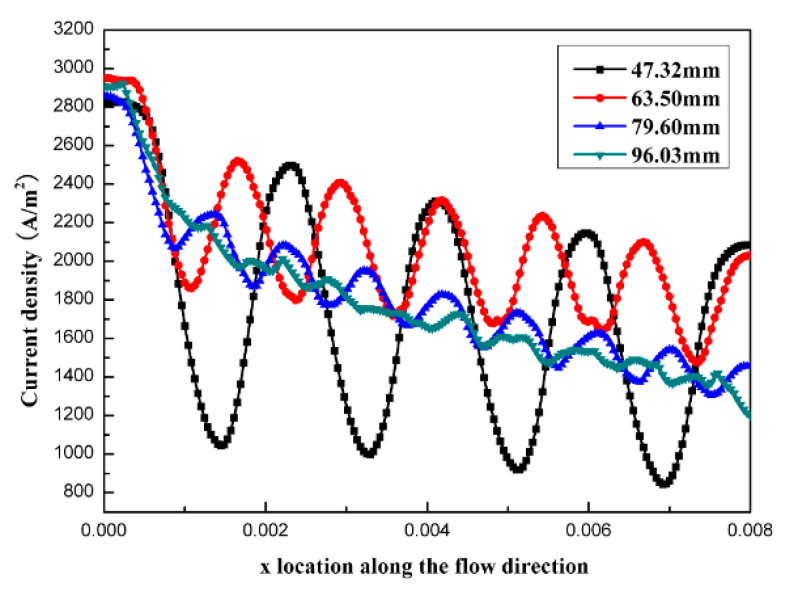
Current density distributions at the catalyst layers along the x direction under the conditions of different open ratios.

Figure 8 shows methanol concentration distributions in the µDMFCs anode flow fields with different channel lengths. With the increment of channel length, the methanol concentration difference between the inlet and outlet increases. Specifically, methanol concentration in the outlet with a length of 96.03 mm is around 150 mol/m^3^ lower than that with a length of 47.32 mm, and might decrease the mass transport efficiency in the outlet of the flow field. On the other hand, increasing the flow field length can improve the methanol mass transport in the diffusion layer. The diffusion dead area under the support ridge can be decreased by increasing the pressure drop, and so the concentration polarization is reduced to a certain extent. Figure 9 shows the current density distributions at the catalyst layers along the x direction (y = 0.004) under the conditions of different channel length. The current density distributions are uneven with the channel length of 47.32 mm. The local current corresponding to the support ridge decreases sharply because this area could have low methanol concentrations. If the over-potential was increased, concentration polarization could occur due to a shortage of fuel. Furthermore, the current density distributions are even, with lengths of 79.60 and 96.03 mm. Though fluctuating a little, the current density average with the channel length of 63.5 mm is higher than the other three.

## 4. Experimental Verification

### 4.1. Fabrication and Assembly

In order to verify the accuracy of the simulation results, µDMFCs with different flow fields were fabricated and assembled. Anode flow field configurations are summarized in Table 5. These configurations are the same as those used in the simulation shown in Section 2.1 Flow Field Design.

A 5-layer MEA with an active area of 0.8 cm × 0.8 cm was fabricated using GDL hot pressing on both sides of the CCM at 130 °C and 4 MPa for 120 s. The 4.0 mgcm^−2^ Pt-Ru anode catalyst, 4.0 mgcm^−2^ Pt cathode catalyst, and Nafion117 membrane as a proton exchange membrane were used to manufacture the CCM by the decal transfer method. The gas diffusion layer (GDL) is carbon paper (TGPH-090, Toray Inc., Tokyo, Japan), which was prepared with the hydrophobic (10 wt.% PTFE for the anode and 30 wt.% PTFE for the cathode) and pore-formed (NH_4_HCO_3_) pretreatment.

The 480 ± 10 μm crystal silicon <100> crystal orientation was used to fabricate the current collector with different patterns using bulk-silicon MEMS technology. First, a 0.8-μm-thick Si_3_N_4_ layer was deposited on a silicon wafer by low pressure vapor deposition. The anode microchannel pattern was formed on the layer by photolithography. Then, the 240 μm deep channels were etched using an anisotropic etching process of 40% KOH at 40 °C. In order to realize a self-breathing cathode, a perforated silicon wafer with a uniform diameter of 0.6 mm was prepared as the cathode. A Ti/Au (0.05 μm/1.0 μm) layer was sputtered on the surface of the current collector to improve current collection and reduce contact resistance.

**Figure 10:**
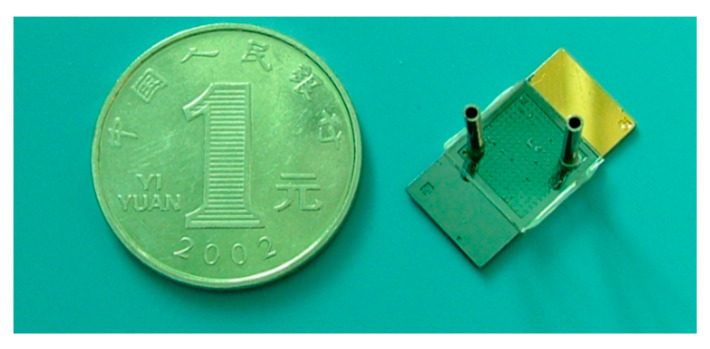
Photograph of an assembled self-breathing micro direct methanol fuel cell (µDMFC).

The MEA was sandwiched between the anode and cathode current collector to form a micro self-breathing direct methanol fuel cell. A clear adhesive was used to complete the seal. The assembly picture is shown in Figure 10. The experiment was carried out at room temperature and atmospheric pressure. A peristaltic pump was used to feed low concentration (1 M) methanol at a feeding rate of 1 mL min^−1^. The performance was measured using an electronic load (N3300A&N3302A, Agilent Technologies, Santa Clara, CA, USA).

### 4.2. Test

**Figure 11:**
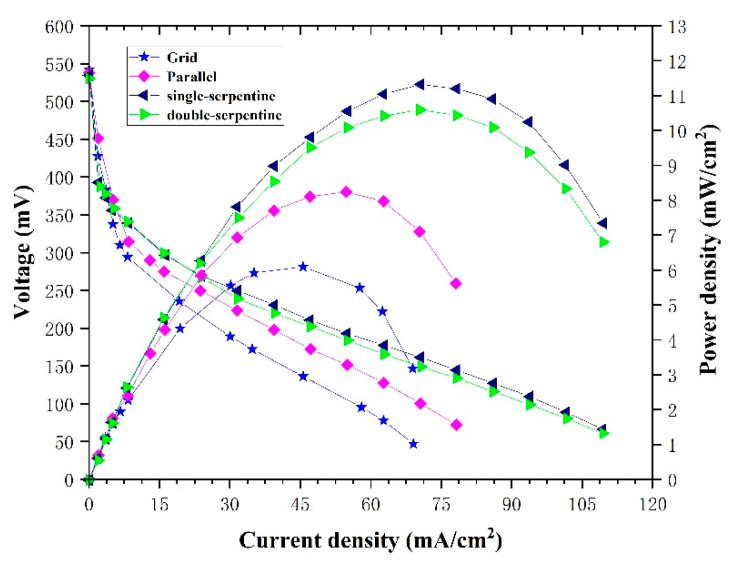
Polarization curves and power density curves of different flow fields under the same experimental conditions.

We first tested the performance of µDMFCs with grid, parallel, single-serpentine and double-serpentine flow fields. The experiments were performed with dilute methanol (concentration 1 M) at the flow rate of 1 mL min^−1^ and at room temperature and atmospheric pressure. Figure 11 shows the performances of the µDMFCs with different anode flow fields. Under different flow field conditions, the cell performance was obviously different, but there is a peak power density. Concentration polarization is the main cause of cell performance decline after peak power density. We found that the micro direct methanol fuel cell with a single-serpentine flow field obtained the best power density of 11.39 mW/cm^2^. However, grid and parallel flow fields showed an earlier power density drop. This indicated that different flow fields have a great influence on the concentration distribution and the single-serpentine flow field has uniform concentration distributions and better carbon dioxide removal. This is consistent with the simulation results.

**Figure 12:**
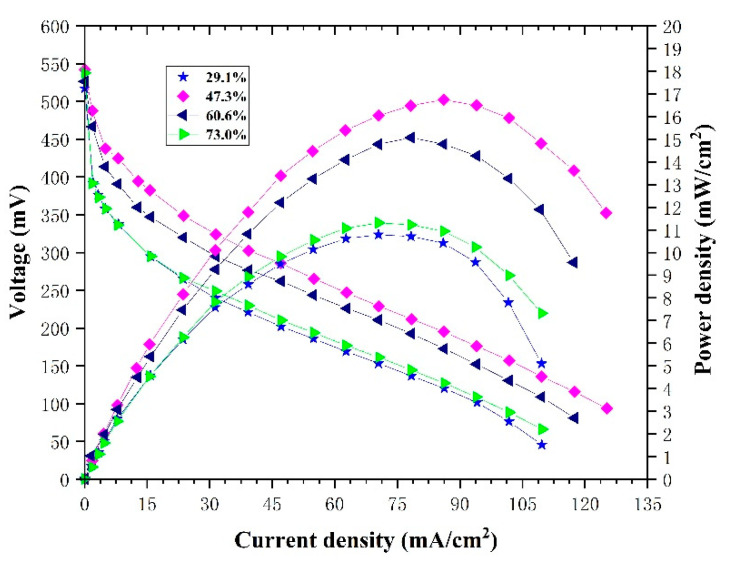
Polarization curves and power density curves of single-serpentine flow fields with different open ratios under the same experimental conditions.

**Figure 13:**
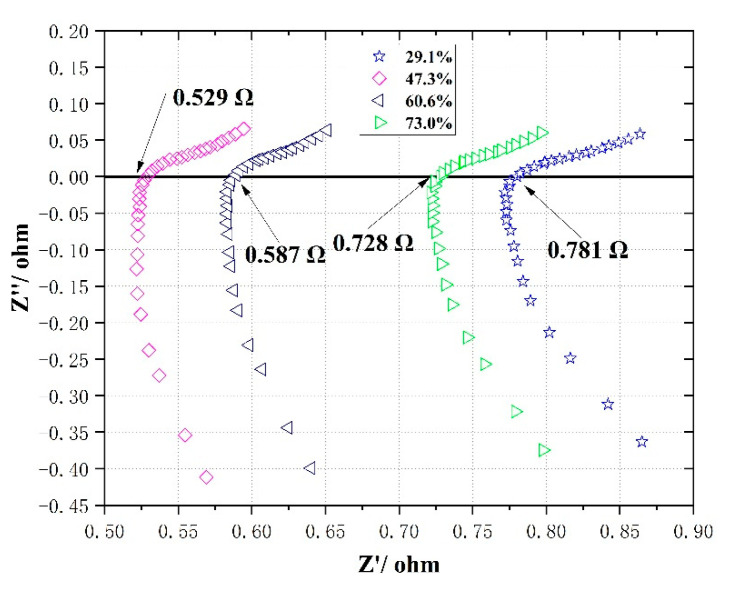
Nyquist plots of single-serpentine flow fields with different open ratios at high frequencies.

From the simulation results for single-serpentine flow fields of different open ratios, it seems that the best performance should be obtained with a 60.6% opening ratio. However, the experimental results in Figure 12 show that the performance decreases in the order of 47.3%, 60.6%, 73.0% and 29.1%, and the maximum power densities are 16.83, 15.13, 11.39 and 10.83 mWcm^−2^, respectively. This seems to differ from the simulation results. In fact, under the condition of the same channel length, the opening ratio determined the channel width. A wider flow channel size will improve the mass transfer between methanol and the diffusion layer, and improve the removal of carbon dioxide. However, an excessively wide channel reduces the width of the rib and increases the contact resistance and methanol crossover. Therefore, due to a channel wider than 29.1% and narrower than 60.6% and 73.0%, the opening ratio of 47.3% achieved the best performance. For a better evaluation of the influence of open ratios on cell performance, we tested the single cell electrochemical impedance spectroscopy (EIS) at a current of 30 mA, with the frequency from 60 kHz to 100 Hz and 10 mV amplitude, as shown in Figure 13. It can be seen that the internal resistance decreases in the order of 47.3%, 60.6%, 73.0% and 29.1%, and was 0.529, 0.587, 0.728 and 0.781 ohm, respectively, which justified the analysis above.

**Figure 14:**
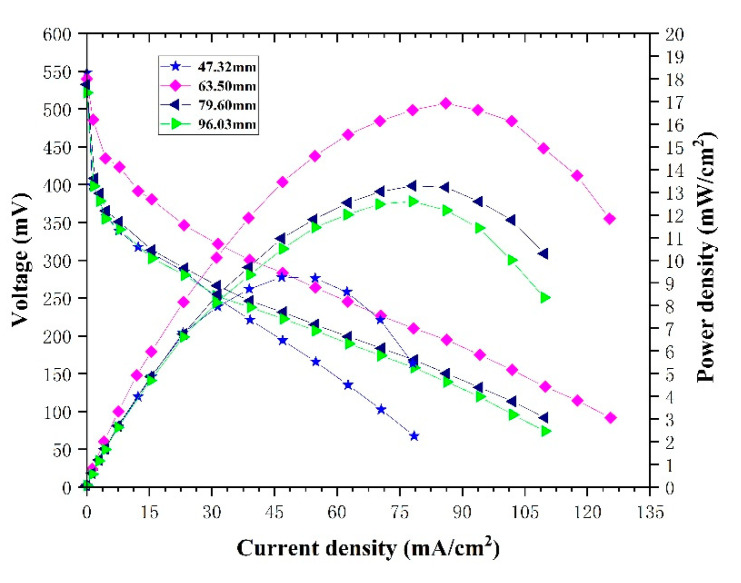
Polarization curves and power density curves of single-serpentine flow fields with different channel lengths under the same experimental conditions.

Figure 14 shows the performance of the µDMFCs with different channel lengths at the same open ratio. The experimental results show that the best power density is 16.83 mWcm^−2^ with the length of 63.50 mm. The result is consistent with the simulation. If the length increases or decreases, the performance degrades. The main reason is that the different length of the channel causes a change of the width of the channel and the rib, resulting in a change of the µDMFC performance.

**Figure 15:**
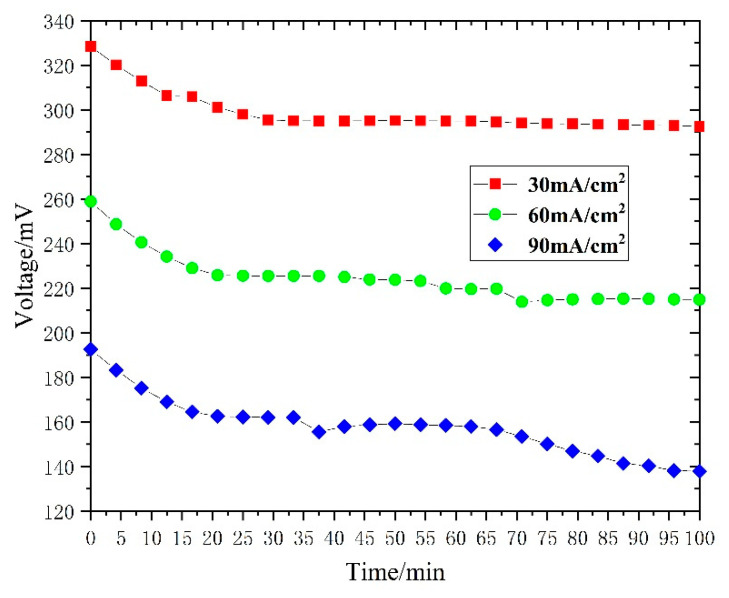
Durability testing results of an air-breathing fuel cell with a single-serpentine flow field at 63.5 mm channel length and 47.3% open ratio at a constant current density of 30, 60, and 90 mAcm^−2^, respectively.

Through the above experiments and analysis, the optimal parameters of single-serpentine flow field were determined as 63.5 mm channel length and 47.3% open ratio. The durability of an air-breathing fuel cell with a single-serpentine flow field at optimal parameters was tested at a constant current density of 30, 60, and 90 mAcm^−2^ respectively. Figure 15 shows the voltage variations of the cell over 100 min. The voltage output of the cell at a constant current density of 90 mAcm^−2^ was more serious, which is mainly because of the fuel starvation at the back end of the cell.

## 5. Conclusions

In this work, the influence of different anode flow fields on the performance of µDMFCs was analyzed by establishing a 3D model of the micro direct methanol fuel cell. A silicon-based direct methanol fuel cell was fabricated and assembled to verify the simulation results. The simulation and experimental results show that compared with grid, parallel and double-serpentine flow fields, the single-serpentine flow field could guarantee uniform distributions and high-efficiency mass transport and the removal of carbon dioxide. In addition, the micro direct methanol fuel cell with a single-serpentine flow field produced a maximum power density of 16.83 mWcm^−2^ at room temperature and atmospheric pressure. For the serpentine flow field, the optimal open ratio of 47.3% and channel length of 63.50 mm were obtained.

## Figures and Tables

**Table 1:** Geometric parameters of four types of flow fields.

Type of Flow Field	Effective Width (mm)	Channel Depth (mm)	Ridge Width (mm)	Effective Area (mm × mm)	Open Ratio (%)
Grid flow field	0.55	0.24	0.69	7.99 × 7.99	72.9
Parallel flow field	0.75	0.24	0.45	7.95 × 7.95	72.9
Single-serpentine flow field	0.8	0.24	0.4	8.0 × 8.0	73.0
Double-serpentine flow field	0.575	0.24	0.25	8.0 × 8.0	73.6

**Table 2:** Geometric parameters of the single-serpentine flow fields with four open ratios.

Open Ratio (%)	Width of Channel (mm)	Channel Depth (mm)	Width of Ridge (mm)	Length of Channel (mm)	Active Area (mm × mm)
29.1	0.3	0.24	0.98	63.68	7.98 × 7.98
47.3	0.5	0.24	0.75	63.50	8.0 × 8.0
60.6	0.65	0.24	0.57	63.32	7.97 × 7.97
73.0	0.8	0.24	0.4	63.20	8.0 × 8.0

**Table 3:** Geometric parameters of the single-serpentine flow fields with four channel lengths.

Length of Channel (mm)	Width of Channel (mm)	Depth of Channel (mm)	Width of Ridge (mm)	Open Ratio (%)	Effective Size (mm × mm)
47.32	0.68	0.24	1.15	47.4	8.0 × 8.0
63.50	0.5	0.24	0.75	47.3	8.0 × 8.0
79.60	0.4	0.24	0.55	47.8	8.0 × 8.0
96.03	0.33	0.24	0.44	47.5	8.03 × 8.03

**Table 4:** Parameters used in the anode model calculations.

Parameter Names	Parameter Values
Faraday constant (F)	96,495 C/mol
Gas law constant (R)	8.314 J/(mol·K)
Standard value ($V_{cell}$)	0.2 V
Standard diffusion coefficient of the methanol in the water ($D_{ref}$)	$6.69\times 10^{-9}\;m^{2}/s$
Electrical conductivity of the electron (σ)	10 S/m
Density of the methanol solution (ρ)	$1000{\;\mathrm{kg}/m}^{3}$
Standard consistence of the methanol ($c_{ref}$)	$1000{\;\mathrm{mol}/m}^{3}$
Exchange current density at the anode ($i_{0}$)	$94.25{\;A/m}^{2}$
Viscosity coefficient of the methanol solution (η)	$0.9\times 10^{-3\;}\mathrm{kg}/\left(m\cdot s\right)$
permeability (κ)	$1.2\times 10^{-12}\;m^{2}$
overvoltage (eta)	0.2 V
porosity (ε)	0.6
Working temperature (T)	293 K
Liquid filling rate ($u_{0}$)	$1.7\times 10^{-8}\;m^{3}/s$
The consistence of methanol ($c_{0}$)	$1000{\;\mathrm{mol}/m}^{3}$
Atmospheric pressure ($p_{0}$)	$1.013\times 10^{5}\;\mathrm{Pa}$
Specific surface area ($S_{a}$)	$10^{7}\;m^{-1}$
Gibbs free energy ($G_{a}$)	$-166.27\times 10^{3}\;J/\mathrm{mol}$
Reaction enthalpy ($H_{a}$)	$-238.66\times 10^{3}\;J/\mathrm{mol}$
Thermal conductivity (k)	1.6 W/(m·K)
Specific heat capacity at standard pressure ($C_{p}$)	2531 J/(kg·K)

**Table 5:** Summary of the experimental anode flow field configurations.

Type of Flow Field	Channel Width (mm)	Channel Depth (mm)	Channel Length (mm)	Ridge Width (mm)	Effective Area (mm × mm)	Open Ratio (%)
Grid flow field	0.55	0.24	- ^1^	0.69	7.99 × 7.99	72.9
Parallel flow field	0.75	0.24	- ^1^	0.45	7.95 × 7.95	72.9
Single-serpentine flow field	0.8	0.24	63.20	0.4	8.0 × 8.0	73.0
Double-serpentine flow field	0.575	0.24	43.30 ^2^	0.25	8.0 × 8.0	73.6
Single-serpentine flow field	0.3	0.24	63.68	0.98	7.98 × 7.98	29.1
	0.5	0.24	63.50	0.75	8.0 × 8.0	47.3
	0.65	0.24	63.32	0.57	7.97 × 7.97	60.6
	0.8	0.24	63.20	0.4	8.0 × 8.0	73.0
Single-serpentine flow field	0.68	0.24	47.32	1.15	8.0 × 8.0	47.4
	0.5	0.24	63.50	0.75	8.0 × 8.0	47.3
	0.4	0.24	79.60	0.55	8.0 × 8.0	47.8
	0.33	0.24	96.03	0.44	8.03 × 8.03	47.5

^1^ The channel length is meaningless in grid flow field and parallel flow field. ^2^ The channel length of the double-serpentine flow field given is the length of a single channel.
